# Predictors of Respiratory Protective Equipment Use in the Norwegian Smelter Industry: The Role of the Theory of Planned Behavior, Safety Climate, and Work Experience in Understanding Protective Behavior

**DOI:** 10.3389/fpsyg.2018.01366

**Published:** 2018-08-08

**Authors:** Øystein Robertsen, Frank Siebler, Martin Eisemann, Marit N. Hegseth, Solveig Føreland, Hans-Christian B. Vangberg

**Affiliations:** ^1^Department of Occupational and Environmental Medicine, University Hospital of North Norway, Tromsø, Norway; ^2^Department of Psychology, UiT—The Arctic University of Norway, Tromsø, Norway; ^3^Department of Occupational Medicine, St. Olavs Hospital, Trondheim University Hospital, Trondheim, Norway; ^4^Department of Geoscience and Petroleum, Norwegian University of Science and Technology, Trondheim, Norway

**Keywords:** safety, behavior, smelting industry, respiratory protective equipment, theory of planned behavior

## Abstract

Previous research has revealed a higher prevalence of respiratory symptoms in Norwegian smelter workers compared to average population controls. Nevertheless, respiratory protective equipment (RPE) is not always used, even in situations with high exposure risk. A questionnaire was distributed to workers in the Norwegian smelting industry to investigate the relationship between psychological factors and self-reported use of RPEs. Response rate was 567/1,253. A scale measuring *attitudes toward behavior* (*ATT*), *subjective norms* (*SN*), *perceived behavioral control* (*PBC*), and *behavioral intention* (*BI*) was constructed based on the Theory of Planned Behavior (*TPB*). Reliability and Confirmatory Factor Analyses partially supported the theoretical structure of the *TPB*-based scale, the Work Experience Measurement Scale (WEMS) and the Short Scale for Safety Climate (SC). A model explaining the relationship between observed variables, latent constructs from *TPB*, WEMS and SC was developed by SEM-analysis. Significant influence on *BI* from *ATT* (β = 0.31 *p* < 0.01)*, SN* (β = 0.36 *p* < 0.01), and *SC* (β = 0.19, *p* < 0.01) emerged. Among the observed variables included, relationship status (β = −0.12 *p* < 0.05), education level (β = 0.09, *p* < 0.05), previously completed respirator fit-testing (β = −0.09, *p* < 0.05) and average hours spent in exposed areas (β = −0.09) *p* < 0.05) had significant influence on behavioral intention. The model explained 48% of the variance in *BI. BI* and *PBC* significantly predicted *PB*, with β = 0.65 and β = −0.06, respectively. Results of this investigation can help facilitate further work and development of health & safety routines within industrial settings.

## Introduction

The metal alloy industry is Norway's largest land-based industry, with a production value of around 7.2 bn EUR per year and is employing ~10,000 (Statistics Norway, 2017).^1^ The production process generate dust, including nano-sized dust, fumes, and gases which pollute the atmosphere and expose workers (Føreland et al., 2008; Johnsen et al., 2008a; Kero et al., 2015, 2017; Kero and Jørgensen, 2016). It has been shown that workplace exposure contributes 10–20% of asthma and Chronic Obstructive Pulmonary Disease (COPD) cases, according to the American Thoracic Society (ATS) (Balmes et al., 2003). Previous research has revealed that workers in the Norwegian smelting industry are more likely to suffer from asthma, COPD, airflow limitation and other respiratory symptoms than the average Norwegian citizen (Bakke et al., 1992; Johnsen et al., 2008b; Søyseth et al., 2011a,b, 2015). COPD is a serious and life-debilitating disease with multiple symptoms and comorbidities. A recent report state that “*Occupational exposures, including organic and inorganic dusts, chemical agents and fumes, are under-appreciated risk factors for COPD development”* (Vogelmeier et al., 2017, p. 577.), and references evidence for increased COPD risk by occupational exposure after smoking was accounted for, and a worse quality of life (Paulin et al., 2015).

In general, work-related deaths in industrial settings were reduced drastically in the twentieth century (CDC, 1999)^2^. For instance, in English industry between 1997 and 2015, fatal injuries decreased by 86%, non-fatal injuries by 77% and annual silicosis deaths have been in a steady decline since the mid-seventies (HSE, 2015)^3^. Over the past century, indoor air quality in Norwegian smelting plants has improved in part due to smarter and more extensive engineering solutions (i.e., automation, ventilation; Føreland et al., 2012). Active campaigns to reduce exposure was initiated by the Norwegian government (Ministry of Health Care Services, 2006, 2013) based on studies in the Norwegian smelting industry (Johnsen et al., 2008c, 2013; Bugge et al., 2011, 2012; Søyseth et al., 2011b).

In addition, construction of exposure free zones such as control-rooms and other barriers prevent worker-exposure through reduced time needed in exposed areas. In a recent survey of Norwegian smelter workers (DeMaskUs study), 98% reported to be exposed to respiratory risk factors during a work-week (Hegseth et al., 2018). Hence, mandatory use of RPE in designated areas is still a common regulatory measure at Norwegian smelters. However, only 28% of workers in the DeMaskUs study reported always using respirators when in mandatory or exposed areas (Hegseth et al., 2018). Furthermore, a study of Norwegian silicon carbide workers reported that only 74% used respirators some- or all of the time when in exposed areas (Føreland et al., 2008). Studies from other industrial settings also show that the use of respiratory protective equipment (RPE) is not always in accordance with regulations or necessity (Salazar et al., 2001; Carpenter et al., 2002; Bryce et al., 2008; MacFarlane et al., 2008; Mitchell and Schenker, 2008; Tam and Fung, 2008; Han and Kang, 2009; Guseva Canu et al., 2013). Thus, inadequate use of respirators does not appear to be exclusive to the Norwegian smelter industry.

The Theory of Planned Behavior (*TPB*) provides a framework to examine the path between beliefs and behavior (Ajzen, 1985, 1991; Fishbein and Ajzen, 2009). The model describes five constructs, *Attitudes toward the behavior (ATT), Subjective Norms (SN), Perceived Behavioral Control (PBC), Behavioral intention (BI)*, and *Previous Behavior (PB)*. *ATT* are the individuals affective and cognitive evaluations of the object in question, SN are evaluations of injunctive and descriptive norms in the social environment and PBC is the degree to which the individual feels he/she possess the skills needed to perform the behavior and if they have autonomy of the behavior. These three components influence *BI*, the individuals' intention to perform a behavior. In addition to *BI, behavior* is influenced by habits, environmental constraints, salience of the behavior, knowledge, and skills (Fishbein and Ajzen, 2009). The *TPB* is an expansion on the Theory of Reasoned Action (TRA) (Madden et al., 1992), which did not include Perceived Behavioral Control. In the present study, the behavior is defined as the reported use of RPEs, and the *TPB* model is used to identify predictors of intention to use RPEs. The *TPB* has previously been used to predict behavior in various settings (Parker et al., 1996; Cordano and Frieze, 2000; Elliott et al., 2003).

Safety climate is a predictor of safe behavior in the workplace, comprising an individual perception of how organization and colleagues influence safety in the workplace (Zohar, 1980; Moore et al., 2005; Clarke, 2006). Safety climate consists of perceptions of social climate, therefore it may overlap to some degree with the *SN* described under the *TPB* as they are both include evaluations of *Subjective norms*. The general safety environment in any industry is expected to influence protective behavior like respirator use.

The Work Experience Measurement Scale (WEMS) is a six-factor survey-tool used to gauge employee attitudes toward management, reorganization, internal work experience, pressure of time, autonomy, and supportive working conditions. These factors comprise a multi-dimensional measure of work experience which can be used for health-promotion in the workplace. If employees evaluation of the organizational climate is related to how employees evaluate respirator use, it could indicate that more general organizational attitudes also predict more specific outcomes such as RPE use (Nilsson, 2010; Nilsson et al., 2013). Accordingly, identifying factors that may influence protective behavior is essential when successful strategies for increased compliance with HSE regulations are devised. To our knowledge this has not been previously investigated in the smelter industry.

The aim of this study was to investigate the influence of the *TPB, SC*, and *WEMS* on intentions to use respirators and reported respirator use in the Norwegian smelting industry.

The current study was reported in two parts. The first part was a pilot study in order to develop and test the theoretical model. The second part consisted of testing the model devised in part one, with the addition of demographics as control variables.

This study assess the following hypotheses:

H1a: Attitudes towards behavior positively influences Behavioral intention.

H1b: Subjective norms positively influences Behavioral intention.

H1c: Perceived control positively influences Behavioral intention.

H2: Safety Climate positively influences Behavioral intention.

H3a: Supportive working conditions positively influences Behavioral intention.

H3b: Internal working experience positively influences Behavioral intention.

H3c: *Autonomy* positively influences *Behavioral intention*.

H3d: Pressure of time positively influences Behavioral intention.

H3e: Management positively influences Behavioral intention.

The previously mentioned hypotheses were tested on the conceptual model shown below.

## Materials and methods

### Demographics

Thousand two hundred and fifty-three questionnaires were distributed and 567 returned (45.25% response rate). The average responder was a 45 year old, in a relationship, male with 19 years of experience in the smelting industry and high-school level education. Most respondents worked in production or maintenance and spent on average 4–6 h per work-shift in a furnace hall. A summary of demographic variables is reported in Table 1.

**Table 1:** Response rates and summary of demographic variables.

**Description**	**Variable**	n^*^	**Response rate**
Questionnaire	Paper (Received/Delivered)	410/710	57.75%
	Electronic (Received/Delivered)	157/533	29.46%
	Total	567/1253	45.25%
		***n***	**Mean (Std. Dev)**
Demographics	Male	493 (86.95%)	
	Female	61 (10.76%)	
	Missing	13 (2.29%)	
	Age	553	45 (13)
	Missing	14 (2.47%)	
	Years experience in smelting industry	552	19 (13)
	Missing	15 (2.65%)	
		***n***	**Percent**
Relationship status	Single	126	22.22
	Married	257	45.33
	Cohabitant	140	24.69
	Widowed	1	0.18
	Divorced/Separated	27	4.76
	Missing	16	2.82
	Total	567	100.00
Number of children	0	163	28.75
	1	83	14.64
	2	171	30.16
	3	94	16.58
	More than 3	39	6.35
	Missing	20	3.53
	Total	547	100.00
Education	Primary/Secondary school	40	7.05
	High school / vocational school	283	49.91
	Private vocational	134	23.63
	University >3 years	49	8.64
	University 3 < years	41	7.23
	Missing	20	3.53
	Total	567	100.00
Previous fit-testing	No	435	76.72
	Yes	110	19.40
	Missing	22	3.88
	Total	545	100.00
Position	Production and maintenance	425	74.96
	Management	91	16.05
	Other	33	5.82
	Missing	18	3.17
	Total	549	100.00
Average hours per day in furnace hall	< 1 h	133	23.46
	1–3 h	149	26.28
	4–6 h	193	34.04
	6–9 h	72	12.70
	10+ h	4	0.71
	Missing	16	2.81
	Total	567	100.00
Smoking	Never	281	49.56
	Sometimes	65	11.46
	1–10 per day	36	6.35
	10–20 per day	41	7.23
	More than 20 per day	1	0.18
	Ex smoker	102	17.99
	Missing	41	7.23
	Total	567	100

*n = number of respondents*.

### Design

The study is a questionnaire-based cross-sectional study comprising demographic and psychological factors.

### Pilot study

#### Questionnaire

A questionnaire containing 176 items was developed to assess aspects of RPE use. The part of the questionnaire used in the current study included demographics variables, a TPB scale, safety climate and WEMS.

#### Demographics

The demographics comprised the following: gender, age, relationship status, status of employment, number of children, level of education, previous participation in RPE fit testing, number of years working in the industry and number of risk factors exposed to. See Table 1 for item wording.

#### TPB factors

One smelting plant was recruited for the development of the questionnaire. This plant (Pilot plant) was not included in the main part of the study. The questionnaire part to measure *TPB* factors (*ATT, SN, PBC, BI*, and *PB*) was developed in several steps:

##### Preparatory work

One of the researchers spent three work-shifts (day, afternoon, and night) following the workers around to gain insight into the job and environment. Using the method described by Fishbein and Ajzen (2009), a questionnaire with an open answer format was sent to 10 employees in three smelters including the Pilot plant, who were asked to report behavioral outcomes, normative referents, and control factors as a foundation for the items generated. Behavioral outcomes are advantages and disadvantages. Normative referents are individuals or groups in the organization that would acknowledge or not acknowledge the use of respirators, most likely or least likely to use respirators while working. Control factors are which factors or circumstances facilitate or debilitate the use of respirators.

##### Focus groups

A group of 28 participants from the Pilot plant were interviewed in seven focus groups using qualitative methods described in the literature (Kitzinger, 1995). The participants were production and maintenance workers. Management were not included in the focus groups to reduce the possibility of social desirability bias. The focus groups were moderated by two members of the research group. The focus groups were conducted as informal conversations, rather than questions and answers. Topics of discussion were health, safety, protective equipment, job tasks, management, social groups, and more. Each interview lasted ~45–60 min. The conversations were tape-recorded with the consent of the participants. The recordings were processed into statements used for item generation.

#### Scale development pilot

Item generation for the pilot questionnaire was based on the open-ended questionnaire and the statements derived from the focus groups. Five to six items were generated for each of the constructs of *TPB*; *Attitudes toward behavior, Subjective norms, Perceived behavioral control, Intention*, and *Previous behavior*. The pilot questionnaire was sent to 31 employees at four of the smelting plants (including the Pilot plant) for assessment, the response rate was 71% (*n* = 22). Following the comments and results, items were modified, removed or added.

A preliminary version of the complete questionnaire was sent to the Pilot plant for further testing. Eighty-five questionnaires were distributed with a response rate of 46% (*n* = 39). Analysis of the preliminary version revealed ceiling effects on most of the *TPB* items. Problematic items were revised to reduce ceiling effects. More specifically, some items were re-worded to increase the conceptual variance between the lowest and highest score. Some items were dropped and some added. Item and scale order was adjusted to make sure that the participants answers on the *TPB* scale were not influenced by their answers on previous items/scales, therefore the *TPB* scale was the first scale after demographics. This would also ensure that if participants lost interest or failed to complete the whole survey, they would be more likely to have completed the *TPB* scale. The initial item pool consisted of 52 items which were discussed with and tested by representatives from the pilot plant resulted in the deletion of 15 items. The finalized version of the *TPB* scale contained 29 items, see Tables A1, A2 in Appendix for item wording. All *TPB* factors scored on a 7-point Likert-scale.

##### A short scale for safety climate (SC).

The scale consists of six questions, see Table A1; three of the six stem from sub-scales of a larger safety climate scale: supervisory performance feedback, worker involvement in safety and coworker behavior norms (Hahn and Murphy, 2008). The remaining three items assess management commitment. The Norwegian version of the SC was developed using translation, back-translation (Sperber, 2004) by independent native speakers and linguistic revision of items by the research group. The questionnaire was sent to five bilingual people who translated it into Norwegian. This translation was then sent out to five different people who translated it back into English to make sure nothing was lost in translation between the Norwegian and the English versions. The research group combined the best translations into the Norwegian version. All safety climate items were scored on a Likert-scale from 1 to 4 where 1 equals “*Completely disagree”* and 4 equals “*Completely agree*.”

##### Work experience measurement scale (WEMS).

The following sub-scales comprise the WEMS, see Table A1 for item wording: Management, Reorganization, Internal work experience, Pressure of time, Autonomy, and Supportive working conditions (Nilsson, 2010; Nilsson et al., 2013). The Swedish version was translated into Norwegian by using the same methodology as the Safety Climate Short Scale. For this study, the Reorganization subscale was excluded due to relevance for the target population and reduction of total questionnaire size. All Work Experience Measurement Scale items were scored on a 1–6 Likert-scale where 1 equals “*Completely disagree”* and 6 equals “*Completely agree*.”

##### Model development.

**Figure 1:**
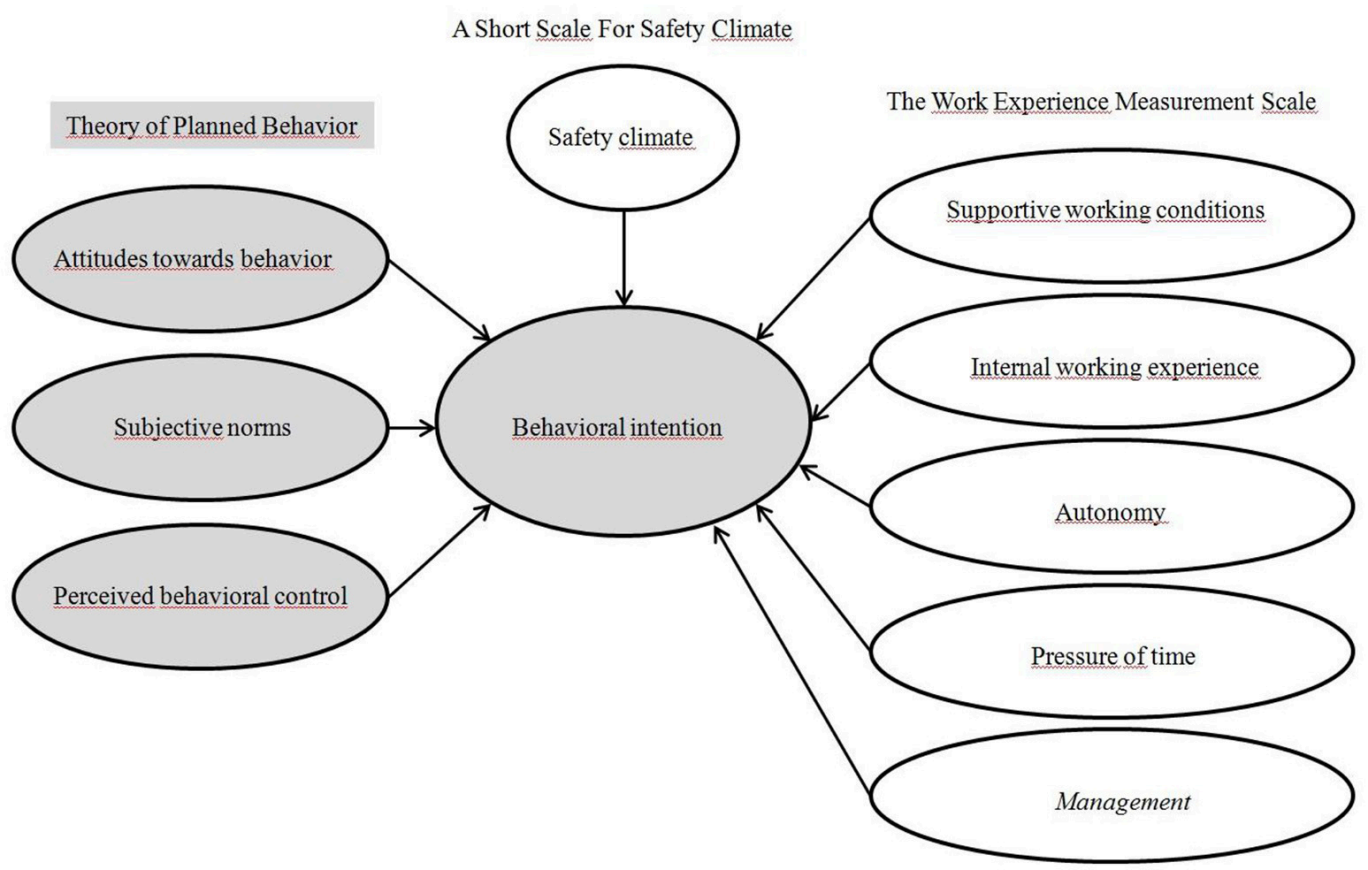
Visual representation of the conceptual model that will be used in the SEM analysis. The Theory of Planned Behavior components are marked with a gray background.

The proposed model is expanded from *TPB* to include SC and the WEMS. The components of *TPB*, SC, and WEMS were expected to influence the intention to use respirators. Due to the cross-sectional nature of the study and no data from previous time-sections, the *PB* construct was left out of the analyzed model. The relationship between *BI* and *PB* was explored by regressing *BI* and *PBC* onto *PB*, as described in the *TPB*. See Figure 1 for visual presentation of the model.

#### Testing the theoretical model

Constructs and indicators are reported in the Appendix with factor loadings, reliability scores and variance extracted.

***Model fit***. The following STATA post-estimation tests were run to assess model fit; Root Mean Squared Error of Approximation (RMSEA), the RMSEA should be lower than 0.05, with a lower bound confidence interval (CI) of < 0.05 and an upper bound CI of < 0.10 (Browne and Cudeck, 1992). An insignificant pclose indicates a tight model fit (Kenny, 2015). The Comparative Fit Index (CFI) (Bentler, 1990) and Tucker-Lewis Index (TLI) (Mehmetoglu and Jakobsen, 2017, p. 309) are both measures of how well the model fit the data, threshold values of >0.90 suggests good fit. Statistics were obtained using STATA post-estimation.

Mehmetoglu and Jakobsen (2017, p. 305) Standardized Factor loadings of <0.6 were discarded stepwise (Chin et al., 1997). Modification indices were assessed to see if any errors between indicators in latent constructs should be allowed to covary (Mehmetoglu and Jakobsen, 2017, p.310).

### Main study

#### Recruitment

In cooperation with The Norwegian Ferroalloy Producers Research Association and silicon-carbide producers, 1,253 workers from 13 smelting plants were recruited to participate in the project. Three plants were silicon carbide producers, four ferro/silicon-manganese, four silicon and two ferro-silicon. Criteria for participating were: (a) working in the smelting industry, (b) and at some point being exposed to respiratory hazards or having to wear RPE, (c) being 18 years or older.

#### Data collection and preparation

Questionnaires were distributed to participating plants either by mail (701 persons at six plants) or online survey (533 persons at seven plants). Data was collected from August 2015 to February 2017. Paper questionnaires were individually enveloped and mailed to each participating plant, where HSE-managers distributed to employees. The envelopes contained a questionnaire, paid-postage return-envelope, information about the project, project participation, and lottery prizes.

Link to the electronic version of the questionnaire was distributed by email addresses provided by HSE-managers. Information about the project, project participation and lottery prizes were included in the email. Two follow-up emails were sent out to encourage completion of the questionnaire.

Returned questionnaires were scanned at the University Hospital of Northern Norway's Clinical Research Department. All data were entered into an SPSS-file for analysis.

Prizes were used to increase likelihood of participation. Participants were eligible to win a gift certificate (6 per plant) of ~80 EUR.

### Ethics

The activities in the current project were considered not to fall under the Norwegian health research legislations by the Regional Committee for Medical and Health Research Ethics. The Norwegian Center for Research Data approved the method for collecting and storing data. The first page of the questionnaire contained information regarding anonymity, voluntary participation, and that participants can at any time withdraw from the study without any consequence. The first page clearly states that by completing the questionnaire, consent is given to participate in the study.

### Data analysis and strategy

The data analysis focused on following the structure of the TPB. As a safeguard against spurious relationships between factors and observed variables, additional interactions were not included in the theoretical model. All demographic items and items used in the TPB latent variables were original items, created by the researchers. The WEMS and Safety climate scale were established prior to the current study.

Statistical analysis were conducted using STATA15 (StataCorp. 2017. *Stata Statistical Software: Release 15*. College Station, TX: StataCorp LLC) and SPSS (IBM Corp. Released 2013. IBM SPSS Statistics for Windows, Version 22.0. Armonk, NY: IBM Corp.). Methods used were descriptive statistics, structural equation modeling (SEM) with post-estimation, validity and reliability assessments and linear regression.

A confirmatory factor analysis was conducted with all items included in their respective factors. An analysis was run with standardized coefficients and maximum likelihood with missing values. Raykov's Reliability Coefficient (RRC) was used as a measure of internal reliability, with a threshold value of 0.7 (Raykov, 1997).

Prior to SEM analysis, “Highschool/vocational school” was collapsed with “Private vocational diploma” and “University >3 years” was collapsed with “University < 3 years.” This was done to make a clearer distinction between “primary/secondary school,” “high-school/vocational school/vocational diploma,” and “university education.” The variable Perceived exposure includes the following; “Which of the following are you exposed to regularly (more than once per week) in your workplace?” There were 15 yes/no options available. A sum score of these was used in the analysis. The more boxes the participants tick off, the more they perceive to be exposed to an element in the working environment. The smoking variable in the analysis was transformed into a “yes/no” response where “no” equals “never” and “Ex smoker.” The remaining options constitute a “yes.”

## Results

### Pilot study

#### Theoretical model evaluation

Post-estimation results did not indicate a good fit for the full theoretical model. (χ^2^ model vs. saturated = 3977.97 and χ^2^ model vs. baseline = 19311.04). The model did better than baseline, but not good enough to indicate that the current number of latent variables and indicators should remain in the model. The root mean squared error of approximation (RMSEA) equaled 0.06, indicating a decent fit. The comparative fit index (CFI) of 0.86 indicated that our model did 86% better than baseline based on χ^2^ and degrees of freedom. The Tucker-Lewis index (TLI) is similar to the CFI, however, it penalizes more complex models. Based on our CFI of 0.86 and TLI of 0.85 we concluded that our model fit was unacceptable, and should be modified (Mehmetoglu and Jakobsen, 2017).

With an average variance extracted below 0.5, it could be seen that the items in *Perceived control* did not adequately measure the same construct. The remaining subscales were convergent. The squared correlation between Perceived control and Behavioral intention was 0.98, indicating that there were items in either perceived control and/or behavioral intention which measured the same construct.

Furthermore, all latent variables in the current model had AVE values below the squared correlations (SC) between latent variables, indicating problems with discriminant validity. A total of nine items were removed from the *TPB* scales according to factor loading criterias. Model fit indices suggested co-varying errors within *ATT* and *SC*, see Table A3. The WEMS scale was completely removed from the model due to low explanatory power.

The revised model was run and re-tested for model fit. Attitudes, Subjective norms, and *Safety Climate* influenced *behavioral intention*, while the *Perceived control* sub-scale was non-significant (*p* > 0.05). Whether the participants were single or not, what level of education they had achieved, if they had previously received fit-testing and how many hours they worked per shift in exposed areas were all significant predictors of their intention to use respirators (Table 2).

**Table 2:** Final model from SEM analysis on effects of latent (TPB) and observed (Demographic) variables on Intention to use respirators (BI) for Norwegian smelter workers.

**Variable**	**Hypothesis**	**SE(B)**	**β**	***z*-value**	***p*-value**	**Conclusion**
Relationship status		0.04	−0.12	−3.36	0.01	
Education level		0.04	0.09	2.34	0.02	
Previously conducted fit-testing		0.04	−0.09	−2.29	0.02	
Average hours spent in exposure per day		0.04	−0.09	−2.22	0.03	
Smoking		0.04	−0.07	−1.83	0.07	
*ATT*	H1a	0.05	0.31	6.87	0.00	Supported
*SN*	H1b	0.06	0.36	6.45	0.00	Supported
*PBC*	H1c	0.05	−0.07	−1.34	0.18	Rejected
*SC*	H1c	0.04	0.19	4.74	0.00	Supported

*R^2^ = 0.48; Root Mean Squared Approximation = 0.04; p of close fit = 1.00; Comparative Fit Index = 0.96; Tucker-Lewis Index = 0.95*.

Fit statistics for the final model were within the threshold with respect to RMSEA for good model fit (RMSEA = 0.04, pclose = 0.1.00) the CFI and TLI were both excellent at 0.96 and 0.95, respectively. All average variance extracted (AVE) values were above squared correlations between factors and all AVE values were above 0.5 indicating no issues with discriminant or convergent validity (Mehmetoglu and Jakobsen, 2017). Raykov's factor reliability coefficients were all acceptable (>0.7).

### Main study

**Figure 2:**
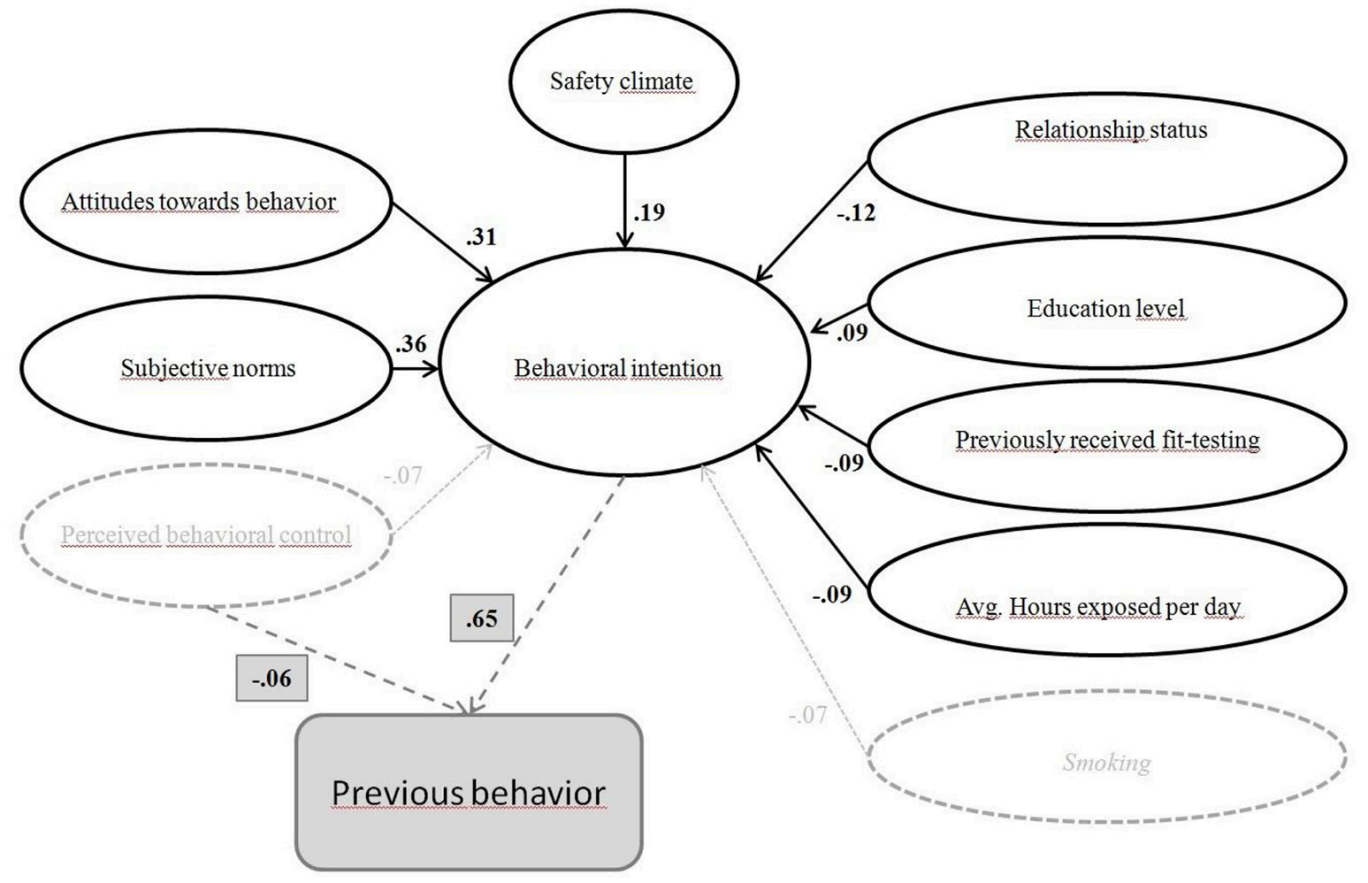
Visual representation of the final model including factor loadings from the SEM analysis described in Table 2 and the regression analysis on PB with standardized beta coefficient (dotted lines). PBC & Smoking did not influence BI (*p* > 0.05) and are therefore faded.

A visual representation of the finalized model, including factor loadings is shown in Figure 2. *ATT* and *SN* corroborated the theorized model (Figure 1) while *PBC* did not influence *BI. Safety climate* was positively associated with *BI*, and the demographic/control variables show that those who were single, had previously participated in fit-testing, and those who spent more hours on average in exposed areas were less likely to intend to use a respirator. Previous behavior was not included in the SEM analysis, as described earlier.

#### Previous behavior

A simple linear regression was calculated to predict *PB* based on *BI* and *PB*. A significant regression equation was found [*F*_(2, 525)_ = 178.42, *p* < 0.00], with an *R*^2^ of 0.49. Participants' predicted *PB* is equal to 2.39 + 0.65(*BI*) −0.06(*PBC*). Standardized betas for *BI* and *PBC* were, respectively, β = 0.65, *p* < 0.01, and β = −0.06, *p* < 0.01.

## Discussion

The pilot study developed a model based on the TPB with some added factors. The main study tested the model on a sample of Norwegian smelter workers to investigate the proposed hypotheses. The described model explained how attitudes, subjective norms, safety climate, and demographic variables predicted intention to use RPE among Norwegian smelter workers. Intention to use RPE and perceived behavioral control predicted the desired behavior (RPE use).

The presented study was conducted on 567/1253 Norwegian smelter workers. 86.95% were male, mean age was 44.5 years with a range of 18–69. 67.55% were non-smokers. The average for Norwegian industries are 76.68% male (Statistics Norway, 2018). A previous study from the Norwegian smelting industry indicate comparable male/female ratio, a slightly lower mean age of 38.6, however inclusion age was different from the current study (20–52 vs. 18–69), which possibly explains the age difference. 51.50% reported to be non-smokers (Johnsen et al., 2008c). 80.59% of the current sample had an education level of primary—high-school/Vocational school (or diploma), compared to 66.70% of the Norwegian population. With respect to these demographics, the respondents in our study seem to be representative for the Norwegian smelter population. The worker population appears stable over time, apart from the reduction in reported non-smokers, which probably mimics the general trend of fewer smokers in the Norwegian society (Norway, 2018).

Participants worked in exposed areas in varying degrees, depending on work-tasks. 98.2% of respondents reported to be exposed to respiratory risk-factors more than once per week (Hegseth et al., 2018). In accordance to the inclusion criteria, employees were excluded if they had positions or work-tasks that did not expose them to risk. Therefore, respirator use was relevant for all respondents.

RPE use was the behavior of interest in this study. Two possible methods of data collection on respirator use was discussed, self-report questionnaires or observational. An observational measure would involve counting or timing employees when using and not using respirators. Observing participants use of respirators could have been problematic if they perceived to be under surveillance. The use of respirators is mandatory for almost all smelter workers, therefore it is not unreasonable to assume that they workers would modify behavior if they either perceive to be observed or actually are observed. This could lead to compromising validity of the results. Therefore, self-report questionnaire was chosen.

According to the *TPB, ATT, SN*, and *PBC* positively influence *Intention and* normally includes the direct influence of intention onto behavior (Fishbein and Ajzen, 2009). The link between intention and previous behavior was challenging to test with the present dataset because the study collected self-reports of behavior retrospectively rather than prospectively. It is difficult to say that something that happens in the present exerts direct influence on something that has occurred in the past. This caused the *Previous behavior* subscale to be omitted from the SEM analysis. However, investigating the influence of participants *BI* on *PB* was one of the main aims of the study. The regression analysis used to explore this relationship, did indeed show a significant association between intention to use respirators *(BI)* and reported RPE use (*PB*), supporting the original structure of *TPB*. The relationship between intention and behavior has been thoroughly established (Sheeran, 2002), supporting the present findings.

Participants' attitudes (*ATT*) toward the use of respirators predicted *BI*. Items in the *ATT* (Table A1) measure how participants evaluate using respirators. The results indicated only slightly positive attitudes toward respirator use (4.40/7), this could be due to issues with comfort and practicalities. Indeed, communication, personal comfort, vision, difficulties breathing, and fatigue has been shown to influence respirator use negatively (Salazar et al., 2001; Baig et al., 2010). This was confirmed in the present sample (DeMaskUs) (Hegseth et al., 2018). It was therefore expected that discomfort and impracticalities would negatively influence *ATT*. Three central COPD traits are *slowly progressing symptoms, a midlife onset*, and *respiratory exposure* (Balmes et al., 2003; Mannino and Buist, 2007). As a result of these traits, it can be difficult to comprehend the importance of protective behavior in the present to avoid problems in the future. The problem of long-term adverse effects is that they are difficult to attribute to a cause, as observed in the context of cigarette smoking, where negative effects are seen later in life (Covington, 1998, p. 211). Hence, practical issues associated with wearing RPE may outweigh their perception of risk related to exposure.

This suggests that to increase RPE use, RPE needs to be as comfortable, practical and easy to use as possible. Personal adjustment is important in order to secure a good fit. Smelting plants could benefit from cooperation with RPE manufacturers in the development of RPE tailor made for the employees in the smelting industry. RPE fit-testing could be beneficial to ensure that the employees have access to functioning RPE. A bonus is that proper quantitative fit-testing can be used as an educational tool to provide employees with real-time examples of what poor and good fit means, in a practical and relatable manner. Being invited to fit-testing should elicit the perception that management is invested in employees safety as well as providing them with proper RPE.

*SN* proved to be the strongest predictor, measuring how participants evaluate other smelter workers' behavior regarding respirator use. The social influence from colleagues appear to be an important contribution respirator use. This result underscores the importance of safety climate. Other studies also found *SN* to be powerful predictors of outcome variables (Chiou, 1998; Fogarty and Shaw, 2010). However, there are discrepancies in the literature regarding this topic, Johnson and Hall (2005) found that behavioral control and intentions were stronger predictors of safe lifting techniques than subjective norms. Also, a review by Armitage and Conner (2001) reported *SN* as a weak predictor of *BI*. Nevertheless, for Norwegian smelter workers, *SN* influence their intentions to use respirators.

*Safety climate* was also a contributing factor of *BI*. The *SC* items (Table A1) addressed perceptions of how health & safety was prioritized and implemented by the organization. The results confirmed our assumption that *SC* is a positive contributing factor in the use of respirators. This is consistent with results from other studies reporting safety climate factors such as organizational support for health and safety, work practices, safety training, and co-worker compliance to be important predictors of respirator use (Nichol et al., 2008; Fukakusa et al., 2011). Extra care should be taken to ensure a positive safety climate, as it is well documented to lead to safety performance (Clarke, 2006). A healthy safety climate has been shown to influence how employees “*…helping coworkers, promoting the safety program within the workplace, demonstrating initiative, and putting effort into improving safety in the workplace” (Neal et al.*, *2000**)*. Indeed, this supports one of our main findings that safety climate is important in order to promote intentions to use respirators, as both *SN* and *SC* influenced *BI*.

As expected, *SN* co-varied with *SC* (data not shown), as they both represented a participant's evaluation of the social environment regarding safety performance. In the present study *SN* and *SC* are related but sufficiently distinct to be two separate constructs. *SN* were descriptive in nature, while *SC* described a more general safety environment. This distinction is also mentioned previous studies (Ehrhart and Naumann, 2004; Fugas et al., 2011, 2012).

The demographic variables influenced *BI* with small effect sizes. Single participants, those who had previously received fit-testing and those who spent more hours in exposure per day influenced *BI* negatively. It may be that people who were married or lived together perceive to have invested more in relationship and material resources, thus evaluating risk differently as indicated Eckel and Grossman (2008) who reported single persons to be more risk-prone than non-singles.

Workers spending more time in exposed areas score lower in *BI*. There are several possible explanations for this observation. Those who spend more time in exposed areas are obligated to use respirators for longer periods of time every day. Wearing a respirator for extended periods of time is uncomfortable. Therefore, it is hardly surprising that those who use respirators for the longest periods of time, report lower intentions. This is in accordance with lower *ATT* scores indicating lower *BI* scores. Another explanation could be that they have adapted to the exposed environment and perceive risk differently. Similar results are found in a study on Californian farmers (Schenker et al., 2002) where a moderate positive association was found between the use of protective equipment and hazardous farm tasks, except working in dust-exposed areas, where the relationship was found to be negative. Another study of this population (Mitchell and Schenker, 2008) reported that greater acreage or time spent in dust exposure was negatively associated with respirator use.

Surprisingly, those who reported previous respirator fit-testing had lower *BI*. Finding a respirator that fits well should intuitively increase *BI* to use it, not reduce it. Indeed, previous studies have shown that respirator fit-testing leads to an increase in respirator use (Salazar et al., 2001; Fukakusa et al., 2011). If employees who have participated in fit-testing have understood why mask-fit is important in reducing exposure, this type of effect should have been positive and strong. However, the study did not ask participants to characterize what kind of fit-testing they had received. Perhaps participants misunderstood what was meant by respirator fit-testing, since an explanation for *fit-testing* was not provided with the questionnaire. No data was collected on when and with what type of respirator fit-testing was done. Fit-testing is a relatively new method in Norway, and not generally implemented in the smelter industry. It is likely that participants did not have previous knowledge of this method. Seen in the context of previous studies, the results from the current study indicates that there might be confounding variables that influence the use of respirators, even when participants have receive fit-testing.

According to our results, participants with higher education were more likely to score higher on intention to use respirators. Individuals with higher education may have a better knowledge of the association between exposure and health effects, or generally more concerned regarding health. A study by (Winkleby et al., 1992) reported education as a significant predictor for smoking, indicating a link between smoking and a reduced likelihood to invest in health over time.

The *previous behavior* factor was an indication of how often the participants had used respirators. The items directly assessed self-reported use according to the *TPB*. *BI* and *PBC* were both found to influence *PB*, explaining 49% of the variance. Participants with positive intentions to use respirators reported higher RPE use. This was expected according to the TPB, confirming the validity of the model. Interestingly, the score for *PB* was higher compared to the *SN*. This suggests that participants evaluated their own behavior as more positive than that of their colleagues. This is supported by Pronin (2008).

Participants with a high perception of control reported less RPE use. According to the *TPB, PBC* should positively influence the *BI*, as well as act as a mediator for *PB* (Ajzen, 1991). The current results indicated that the items measuring *PBC* did not accurately reflect the theoretical structure, possibly due to measurement error or confounding variables. The current model supports the TRA, where *ATT* and *SN* influence *BI*, rather than the *TPB* where *PBC* is included. Interestingly, in the regression analysis of the relationship between *BI* and *PB, PBC* significantly influenced *PB*, albeit with a small negative effect size, suggesting that the *PBC* factor shares some variance with the *ATT* and *SN* factors in the SEM analysis. The proposed model where *BI* was influenced by *ATT, SN, PBC, SC*, and *WEMS* did not fit the data with acceptable parameters, rejecting the researchers hypothesis that these factors would explain participants intentions to use RPE. As the analysis showed, WEMS and PBC did not work as intended in this circumstance. The WEMS measures a more general picture of work experience which may not be relevant to intentions to use respirators. How employees feel about autonomy, meaningful work-tasks, job satisfaction and management attitudes might not influence RPE intentions at all.

Age, number of children, experience in years, perceived exposure, and smoking were all factors that were thought likely to influence intention to use respirators, but did not emerge as predictors. Smoking can be said to indicate a negative relationship with intention, although this is not statistically significant.

A possible explanation for differences between individuals that have not been accounted for in this study, but should be mentioned, is the perspective of the Transtheoretical model as a framework for describing behavior change. The model describes behavioral change to evolve through different stages. In the context of the current study, individuals at the lowest level of change could be unaware, not knowing that they ought to change their behavior, to the top level, where they have concluded that they will not ever return to their old, perhaps unhealthy way of behaving. The steps in-between involve recognition of problem, contemplate changing, preparation and then actual behavior change, followed by a period of maintenance (Prochaska et al., 2002). Future studies should investigate if this is the case.

Human resources personnel could use the results of this study to further develop safety cultures and climates in the smelting industry. The developed *TPB* scale and the Short Scale for Safety Climate should be validated further to investigate and map employees' attitudes toward RPE and their thoughts on safety climate. Validated instruments can enable organizations to discover and monitor possible issues regarding protective behavior that can be managed through strategic health & safety work.

The present study applied a theory previously used in e.g., the field of sports, health, and education. This theory was applied to in the Norwegian smelting industry, thereby adding to the pool of applied TPB research explaining the precursors of behavior.

## Limitations

In all studies concerning human behavior, using questionnaires as the main source of data presents some innate limitations. The questionnaire in the current study was designed for this purpose and has not been validated elsewhere. The items may in some cases not have measured the intended theoretically structures, as discussed above.

Also, the behavior of interest (RPE use) is governed in part by rules and regulations of each plant. There is a clear expectation from the employers that employees take steps to protect themselves accordingly. Whenever participants respond to a question where the “correct” answer is obvious, they may be influenced by social desirability bias, i.e., the employer expects the employee to use RPE.

The process of creating items and scales to include in analysis is subject to limitations by those who take partake in the task. Experience dealing with participants and conducting focus groups, for instance, are two areas of method which can be unintentionally influenced by the researchers. The study could be subject to a range of unidentified confounding variables. The questionnaire consisted of 174 items total, and would take between 25 and 45 min to complete, depending on literacy. A large questionnaire size could influence response rate negatively by respondents dropping out or not responding at all.

A response rate of 45.25% is acceptable considering the study design and method of data collection. However, a non-response bias of over 50% should be considered when interpreting the results. There were no data collected from non-respondents. As a result of this, there could be a systematic difference between the sample population and the population. However, comparing the respondent population to the population described in previous studies from the Norwegian smelter industry (Johnsen, 2008), indicated that the responders were a representative sample. The electronic questionnaire distributed by e-mail achieved a lower response rate than the paper version, as expected. Follow-up requests to complete questionnaires were sent to participants to reduce the drop in response rate. To optimize response rate and ensure commitment, the researchers conducted meetings with key HR personnel and management in the planning phase. Meetings were held at the 6 plants that received paper questionnaires but not at all of the plants that received electronic questionnaires, which may explain the lower response rate. Another explanation for the low response rate on the electronic questionnaire could be that participants did not have computers easily available at work, or that they did not check their work e-mail often.

This study employed a number of demographic variables as controls (Table 1). As the number of variables increase, so does the chance of discovering a significant result by chance. Therefore, it is possible that some of the results in the present study are by chance, caused by the number of variables included in the analysis. Nevertheless, we chose to include the variables as they were expected to have an effect on intention and behavior. Additionally, the current study focused on direct effects of independent variables on behavioral intention. In future research the investigation of any interaction, mediation, or moderation effects between the independent variables on behavioral intention, should be of interest.

## Summary

The current study on 567 Norwegian smelter workers revealed that *Attitudes toward* behavior (*ATT*), *Subjective norms* (*SN*), *Safety climate* (*SC*), education level, relationship status, previous respirator fit-testing, and average hours spent in exposed areas are predictors of the workers intention to use RPE (*Behavioral intention, BI*). *Behavioral intention* and *Perceived behavioral control* (*PBC*) in turn predicts *Previous behavior* (*PB*), in this study defined as RPE use.

## Author contributions

ØR: design, data collection, analyses, writing. FS: design, review, analyses. ME: design, review. MH and H-CV: design, data collection, review, writing. SF: data collection, review, writing.

### Conflict of interest statement

The study was partially funded by the Norwegian smelting industry. The authors declare that the research was conducted in the absence of any commercial or financial relationships that could be construed as a potential conflict of interest.

## Footnotes

^1^https://www.ssb.no/energi-og-industri/statistikker/sti/aar-endelige

^2^https://www.cdc.gov/mmwr/preview/mmwrhtml/mm4822a1.htm

^3^http://www.hse.gov.uk/statistics/history/historical-picture.pdf
