# Supplementary material for: Predictors of Respiratory Protective Equipment Use in the Norwegian Smelter Industry: The Role of the Theory of Planned Behavior, Safety Climate, and Work Experience in Understanding Protective Behavior
Source: Front Psychol. 2018 Aug 8;9:1366. doi: 10.3389/fpsyg.2018.01366 (PMC6092595; doi:10.3389/fpsyg.2018.01366)
Supplement: Supplementary file 1 [file Table_1.docx]

Appendix

Table A1. *Measurement model TPB scales, SC and WEMS* n = 542

| Constructs and indicators | Factor Loadings | Raykov's composite reliability | Variance Extracted | Mean | Std. Dev. |
| --- | --- | --- | --- | --- | --- |
| ***Attitudes**** |  | .92 | .56 |  |  |
| 16.1 Regularly using a respirator during the next work-week would be ... |  |  |  |  |  |
| a … Very harmful - Not harmful at all. | .20 |  |  | 6.45 | 1.21 |
| b … Very uncomfortable - Very comfortable. | .83 |  |  | 4.10 | 1.70 |
| c … Very impractical - Very practical. | .86 |  |  | 4.17 | 1.83 |
| d … Very cumbersome - Not cumbersome at all. | .77 |  |  | 4.79 | 1.75 |
| e … Not desirable at all - Very desirable. | .78 |  |  | 4.66 | 2.10 |
| f … Very annoying - Not annoying at all. | .88 |  |  | 4.13 | 1.85 |
| g … Hindering - Not hindering at all. | .83 |  |  | 4.17 | 1.89 |
| h … Very exhausting - Very easy. | .69 |  |  | 4.80 | 1.67 |
| ***Subjective norms**** |  | .86 | .51 |  |  |
| 16. 4. Most people I know appreciate that I wear a respirator at work. | .60 |  |  | 5.64 | 1.83 |
| 16. 5. Most workers like me always use a respirator at work. | .92 |  |  | 4.04 | 2.16 |
| 16. 6. My family appreciates that I wear a respirator at work. | .52 |  |  | 5.83 | 1.76 |
| 16. 7. All my colleagues always wear respirators while at work. | .89 |  |  | 3.62 | 2.24 |
| 16. 8. There aren't many workers who adhere to the respirator policy during a work-week. | .29 |  |  | 3.78 | 1.92 |
| 16. 9. My colleagues at the plant usually wear a respirator at work. | .83 |  |  | 4.51 | 1.99 |
| ***Perceived control**** |  | .04 | .19 |  |  |
| 16. 13. It's up to me whether or not I use the respirator during a work-week. | .29 |  |  | 4.53 | 2.44 |
| 16. 14. I adjust the respirator correctly every time I put it on. | -.41 |  |  | 5.67 | 1.55 |
| 16. 15. I am in charge of which situations I use the respirator in during a work-week. | .41 |  |  | 4.46 | 2.42 |
| 16. 16. I can't use a respirator in all situations during a work-week. | -.36 |  |  | 3.08 | 2.23 |
| 16. 17. I use the respirator in all work-situations even though it is demanding. | -.65 |  |  | 5.37 | 1.74 |
| ***Behavioral intention**** |  | .84 | .51 |  |  |
| 16. 19. I won't be using the respirator during the next work-week. | .42 |  |  | 2.28 | 2.01 |
| 16. 20. I'm going to use the respirator even though it makes it harder to breathe. | .70 |  |  | 5.27 | 1.89 |
| 16. 21. I am going to use the respirator in all required situations next work-week. | .69 |  |  | 5.56 | 1.82 |
| 16. 22. I am going to use the respirator even though it is warm and uncomfortable. | .86 |  |  | 5.68 | 1.68 |
| 16. 23.I am constantly going to use the respirator in the furnace hall/exposed areas next work-week. | .64 |  |  | 4.19 | 2.39 |
| 16. 24. I am going to use the respirator even though it is impractical. | .87 |  |  | 5.38 | 1.78 |
| ***Safety climate***** |  | .89 | .56 |  |  |
| 17.1. New employees learn quickly that they are expected to follow good health and safety practices. | .62 |  |  | 3.42 | .78 |
| 17.2. Employees are told when they do not follow good health and safety practices. | .63 |  |  | 3.24 | .86 |
| 17.3. Workers and management work together to ensure the safest possible conditions. | .82 |  |  | 3.18 | .86 |
| 17.4. There are no shortcuts taken when worker health and safety are at stake. | .82 |  |  | 3.15 | .88 |
| 17.5. The health and safety of workers is a high priority with management where I work. | .87 |  |  | 3.24 | .86 |
| 17.6. I feel free to report safety problems where I work. | .69 |  |  | 3.42 | .82 |
| ***Supportive working conditions****** |  | .88 | .52 |  |  |
| 31.1. We encourage and support each other at work. | .72 |  |  | 4.76 | 1.07 |
| 31.2. There's a good atmosphere at my workplace. | .77 |  |  | 4.87 | 1.09 |
| 31.3. I feel we have well-functioning routines at my workplace. | .80 |  |  | 4.66 | 1.06 |
| 31.4. I get feedback on my work. | .65 |  |  | 3.85 | 1.40 |
| 31.5. I thrive at my workplace. | .71 |  |  | 5.08 | 1.03 |
| 31.6. It feels like my employer invests in my health. | .66 |  |  | 4.39 | 1.26 |
| 31.7. I get advice and help from others when I need it. | .74 |  |  | 4.88 | 1.09 |
| ***Internal working experience****** |  | .85 | .50 |  |  |
| 31.8. My work feels meaningful. | .71 |  |  | 4.80 | 1.12 |
| 31.9. I feel like I am developing myself through my work. | .79 |  |  | 4.34 | 1.28 |
| 31.10. My work is varied. | .76 |  |  | 4.59 | 1.28 |
| 31.11. I feel that I am doing the job I am educated to do. | .76 |  |  | 4.40 | 1.47 |
| 31.12. I go to work with joy. | .42 |  |  | 4.74 | 1.19 |
| 31.13. My work is challenging. | .69 |  |  | 4.41 | 1.30 |
| ***Autonomy****** |  | .86 | .60 |  |  |
| 31.14. I decide when to do different work-tasks. | .78 |  |  | 3.59 | 1.51 |
| 31.15. I decide what gets done in my job. | .84 |  |  | 3.31 | 1.50 |
| 31.16. I decide how my work is done. | .78 |  |  | 3.93 | 1.52 |
| 31.17. I decide my work pace. | .69 |  |  | 4.17 | 1.47 |
| ***Pressure of time****** |  | .81 | .59 |  |  |
| 31.18. I complete my work-tasks during normal working hours without any pressure of time. | .80 |  |  | 4.17 | 1.42 |
| 31.19. Usually I have time to finish one work-task before I have to start the next. | .85 |  |  | 4.08 | 1.49 |
| 31.20. I rarely have to stay at work past normal working hours. | .63 |  |  | 4.64 | 1.48 |
| ***Management****** |  | .92 | .66 |  |  |
| 31.21. My manager is available when I need something. | .72 |  |  | 4.55 | 1.33 |
| 31.22. My manager does a good job creating interest and engagement around work-tasks. | .88 |  |  | 3.95 | 1.38 |
| 31.23. My manager contributes to a fair delegation of work-tasks. | .84 |  |  | 4.03 | 1.40 |
| 31.24. My manager discusses issues with the work-force before an important decision is made. | .85 |  |  | 3.97 | 1.49 |
| 31.25. My manager has the ability to make his/her own decisions when needed. | .76 |  |  | 4.64 | 1.36 |
| 31.26. My manager ensures that information regarding the goals and visions of the business is available to the workforce. | .81 |  |  | 4.31 | 1.33 |

Note: *Measured on 1-7 Likert.

**Measured on 1 - 4 Likert.

***Measured on 1 - 6 Likert.

Items 16.4, 16.6, 16.9, 16.13-16.17 and 16.19-16.24 were scored *"Completely disagree - Completely agree"*

Items 16.5, 16.7 and 16.8 were scored *"Very unlikely - Very likely".*
