# Supplementary material for: Predictors of Respiratory Protective Equipment Use in the Norwegian Smelter Industry: The Role of the Theory of Planned Behavior, Safety Climate, and Work Experience in Understanding Protective Behavior
Source: Front Psychol. 2018 Aug 8;9:1366. doi: 10.3389/fpsyg.2018.01366 (PMC6092595; doi:10.3389/fpsyg.2018.01366)
Supplement: Supplementary file 2 [file Table_2.docx]

Table A2. Previous behavior variable n = 532

| Construct and indicators | Factor loading | Raykov's composite Reliability | Average Variance extracted | Mean | St.dev |
| --- | --- | --- | --- | --- | --- |
| During the previous work-week I have... |  | .86 | .62 |  |  |
| 16.25 Always used a respirator in exposed areas | .76 |  |  | 5.30 | 2.00 |
| 16.26 Always used respirator in warranted situations | .90 |  |  | 5.86 | 1.65 |
| 16.27 Always changed respirator /filter when needed | .72 |  |  | 5.68 | 1.71 |
| 16.28 Always used a respirator according to regulations | .73 |  |  | 5.90 | 1.50 |
