# Supplementary material for: Predictors of Respiratory Protective Equipment Use in the Norwegian Smelter Industry: The Role of the Theory of Planned Behavior, Safety Climate, and Work Experience in Understanding Protective Behavior
Source: Front Psychol. 2018 Aug 8;9:1366. doi: 10.3389/fpsyg.2018.01366 (PMC6092595; doi:10.3389/fpsyg.2018.01366)
Supplement: Supplementary file 3 [file Table_3.docx]

Table A3. Modification indices.

| Factor | Item pairs | Modification Index |
| --- | --- | --- |
| *ATT* | 16.1b*16.1c | 29.12 |
|  | 16.1c*16.1f | 16.16 |
|  | 16.1c*16.1h | 13.53 |
|  | 16.1d*16.1g | 17.44 |
|  | 16.1d*16.1h | 33.26 |
|  | 16.1f*16.1g | 28.20 |
|  | 16.1g*16.1h | 11.84 |
| *SC* | 17_1*17_2 | 82.80 |
|  | 17_1*17_5 | 25.54 |
|  | 17_2*17_5 | 18.77 |
|  | 17_5*17_6 | 18.00 |
